# Editorial: Insights in pediatric urology: 2022

**DOI:** 10.3389/fped.2025.1544314

**Published:** 2025-01-14

**Authors:** Marco Castagnetti, Miguel A. Castellan, Ezekiel E. Young

**Affiliations:** ^1^ Department of Urology, University Hospital of Padova, Padova, Italy; ^2^ Department of Urology, Bambino Gesù Children’s Hospital (IRCCS), Rome, Italy; ^3^ Department of Urology, Joe DiMaggio Children’s Hospital, Hollywood, FL, United States; ^4^ Department of Urology, Nicklaus Children’s Health System, Miami, FL, United States; ^5^ Leonard M. Miller School of Medicine, University of Miami, Miami, FL, United States; ^6^ Department of Urology, University at Buffalo, Buffalo, NY, United States; ^7^ Department of Urology, John R. Oishei Children’s Hospital, Buffalo, NY, United States

**Keywords:** editorial, pediatric urology, ureteropelvic junction obstruction (UPJO), pyeloplasty, testicular torsion, cloacal malformations, nocturnal enuresis

**Editorial on the Research Topic**
Insights in pediatric urology: 2022

This collection of articles has a wide selection of studies that will contribute to further progress in Pediatric Urology. Managing these various pediatric urological topics contributes to the community's knowledge.

Two articles described the current management of pyelo-ureteral junction obstruction. Zarfati et al. from Italy addressed the issue of the management of poorly functioning obstructive kidneys with a specific focus on conservative management. Between 2009 and 2021, the authors managed 17 patients with unilateral obstructive hydronephrosis and a differential renal function (DRF) below 20%. Function recovery after a trial of internal urinary diversion with a double J stent was quite exceptional, ranging 9% to 12%, up to a maximum DRF of 28%. No difference was observed in the outcome between patients managed surgically and conservatively after stent removal. These data support the idea that, in many of these patients, the outcome is unrelated to the kind of surgical repair elected, and surgical treatment and invasive procedures might be avoided (1).

Unfortunately, no criteria can help select the cases that could benefit from treatment. Pérez-Marchán and Pérez-Brayfield, from Brazil, conducted a retrospective cohort study to compare laparoscopic pyeloplasty (LP) and robot-assisted pyeloplasty (RALP) with at least 3 years of follow.

From 2008 to 2019, 86 patients were treated, 44 (51.1%) underwent LP, and 42 (48.8%) a RALP. The two procedures showed comparable operative time, length of stay, success rate, and post-operative hydronephrosis grading. RALP was more expensive, due to the costs of the supplies. This study confirms that both approaches are valuable, and the choice depends on surgeon preference and finances (2).

Surgical reconstruction for cloacal malformation with a long common channel (>3 cm) can be technically challenging. A manuscript from Seoul, Republic of Korea (Gang et al.), reported the use of MRI-based 3D imaging and printing before correcting this complex malformation. Three-D reconstruction and printing have been reported to provide accurate visualization and quantification of the size and spatial relationship of each cloacal structure in the pelvis (3). Authors believed this approach has facilitated the accurate spatial anatomy of the cloacal systems and has assisted in selecting the appropriate surgical procedure. They expected that 3D images based on MRI cloacagram using saline as a contrast agent may eventually replace endoscopic examinations during surgical planning for children with complicated cloaca. In this manuscript, they demonstrated that the use of MRI-based 3D printing, using saline as a contrast agent, during surgical planning of cloacal malformation correction, led to a successful postoperative outcome.

Xu et al., from Wenzhou and Wuhan, China have studied cases of testicular torsion in which the pre-operative ultrasound demonstrated preserved testicular blood flow. They identified seven patients who had preserved blood flow on ultrasound, but who were still taken for surgical exploration, which demonstrated findings consistent with torsion. Retrospectively studying these seven “false-negative” patients, the authors studied their pre-operative ultrasound findings, intending to identify ultrasound findings that may help diagnose true torsion in patients with preserved Doppler flow. They reported the following ultrasound characteristics for such patients: increased testicular volume as compared to the asymptomatic testis (100%), oblique lie (57.1%) rather than upright position, heterogeneous parenchymal echotexture (71.4%), enlarged epididymis and increased epididymal blood flow (85.7%), hydrocele (71.4%), and “whirlpool sign” (100%). The authors suggest that these ultrasound findings should prompt strong consideration for surgical exploration, regardless of preserved Doppler flow. Just as the TWIST score has aimed to predict the probability of testicular torsion using clinical non-radiographic assessment (4), data such as these authors present may also be able to help us predict the probability of true torsion in those rare cases where the ultrasound demonstrates preserved doppler flow.

In an additional Chinese manuscript, Li et al., from Shanghai University, have performed a bibliometric and visual analysis to study the body of published literature on nocturnal enuresis. Bibliometric analysis is a computer-assisted review methodology that uses bibliographic data to analyze trends in research (5). Using data retrieved from the Web of Science core collection, they analyzed information about 1,111 studies published between 1982 and 2022. They report the analysis demonstrated that the United States was the most prolific country for such research while Ghent University was the single most influential institution and Rittig Soren was the single most prominent scholar. The authors describe that over the 40 years examined, there has been ongoing growth each year in the amount of nocturnal enuresis literature. They use several different bibliographic techniques to generate visual analyses of literature. They report that the ‘current research hotspots include treatment modalities, epidemiological investigations, and explorations of pathogenesis. They report that the potential research hotspots include clinical research, adenoidectomy, aquaporin-2, and response inhibition. Finally, they report that standardization of terminology, and pathologies of polyuria and sleep disorder are at “the forefront of research.”
